# Antibiotic prescribing intensity and community UTI resistance: a cross-sectional ecological study

**DOI:** 10.3399/BJGPO.2023.0248

**Published:** 2025-03-26

**Authors:** Lucy McDonnell, Mark Ashworth, Peter Schofield, Stevo Durbaba, Patrick Redmond

**Affiliations:** 1 Department of Population Health, King’s College London, London, UK; 2 Department of General Practice, Royal College of Surgeons, Dublin, Ireland

**Keywords:** antibiotic, anti-bacterial agents, prescribing, resistance, urinary tract infections, primary health care

## Abstract

**Background:**

Antibiotic overuse is associated with antimicrobial resistance (AMR). It is unclear whether community AMR is driven by overall antibiotic use or by high levels of repeated (intense) use by individual patients.

**Aim:**

To determine the association between high antibiotic prescribing intensity (all antibiotic prescriptions; any indication), and rates of urinary tract infection (UTI) resistance among patients within small communities.

**Design & setting:**

Repeated cross-sectional ecological analysis of geographical areas (population averaging 1500) from 2012–2015 using anonymised primary care data and urine cultures.

**Method:**

For each area, we compared the percentage of patients prescribed antibiotics who received≥5 prescriptions over 3 years or≥4 prescriptions over 1 year, with subsequent or same year UTI resistance rates. We also compared Average Daily Quantities (ADQ) of all antibiotics prescribed, with UTI resistance rates, per year, per area. Results were adjusted for covariates and analysed at area level using mixed effects logistic regression.

**Results:**

Of 196 513 patients prescribed antibiotics in 69 areas, 16% were prescribed intensively (≥5 prescriptions in 3 years), receiving almost 30% of prescriptions. Of 12 308 confirmed UTI specimens (80% *Escherichia coli*), 65% were resistant to at least one antibiotic (amoxicillin; cefalexin; ciprofloxacin; trimethoprim; nitrofurantoin). We found no significant association between high intensity ‘any’ antibiotic prescribing (same year or 2 preceding years) or overall ‘any’ antibiotic prescribing (same year) and UTI resistance.

**Conclusion:**

We found no relationship between concurrent high intensity ‘any’ antibiotic prescribing, and UTI resistance rates in small urban communities, pre-COVID-19. Individual patient use of multiple antibiotics, even at high intensity, may not be an independent risk factor for community UTI resistance.

## How this fits in

Few studies have investigated the effect of intensity of antibiotic prescribing on antimicrobial resistance (AMR). We found no evidence that high intensity prescribing of multiple antibiotics to individual patients within the past 3 years was associated with increased UTI resistance rates in small urban communities. Our study suggests that intensive prescribing of multiple antibiotics for individual patients in primary care, may not be any more detrimental to resistance rates, than overall community antibiotic prescribing.

## Introduction

Overprescribing of antibiotics is a modifiable risk factor for antimicrobial resistance (AMR),^1,2^ with most antibiotic prescribing occurring in primary care.^3^ Although there is evidence that overall antibiotic prescribing in primary care has been decreasing since 2012, the most recent English Surveillance Programme for Antimicrobial Utilisation and Resistance (ESPAUR) report showed that antibiotic prescribing is starting to increase again.^3^ Antibiotic regimens are often prescribed for longer than recommended guidelines^4^ and are not evenly distributed within communities. There is variation in prescribing by geographical area and prescribing to individuals within areas.^5^ In a subset of 385 UK primary care practices, fewer than 10% of patients were prescribed 50% of antibiotic prescriptions, with those patients receiving at least five prescriptions over 3 years.^6^

There is limited evidence regarding whether community AMR is associated with overall use of antibiotics in general, or by high levels of repeat prescribing to individual patients (intensive use). A large US-based cross-sectional study (examining 99.8 million antibiotic outpatient prescriptions) concluded that first-time use of specific antibiotics was more strongly associated with resistance than repeated use, when examining specific pathogen–antibiotic combinations.^7^ The same data, however, were re-analysed by other researchers who found that when examining ‘any’ antibiotic use, intensive use (repeated use by individual patients) was more often positively associated with high resistance than first use.^8^ It is unclear therefore whether it is more effective to reduce overall antibiotic exposure or high levels of repeat prescribing to individuals to tackle AMR.

Urinary tract infections (UTIs) account for approximately 15%–20% of antibiotics prescribed in primary care; the second most common infection for which antibiotics are prescribed.^9^ Resistance rates in England vary according to organism and antibiotic and by geographical area.^3^ Although most UK antibiotic prescriptions are for respiratory infections,^10^ there is rarely microbiological confirmation, which makes studying community resistance patterns impractical. In comparison, urine samples are often sent for culture and sensitivity, allowing analysis of antibiotic susceptibility. Clinical guidelines, however, advise only sending samples for high-risk groups, so it is likely that the rate of resistant samples analysed in the laboratory is overestimated.^11^ Nevertheless, recent studies suggest that routine laboratory data are adequate to determine estimates of non-susceptibility to key antimicrobials in community-acquired UTIs.^12^

The aim of this study was to determine the association between intensive prescribing of multiple antibiotics and rates of UTI resistance to five commonly used antibiotics within small geographical areas. We defined intensive antibiotic prescribing as the prescription of≥5 antibiotics within 3 years for an individual patient. We defined overall antibiotic prescribing as the Average Daily Quantity (ADQ) of each antibiotic prescribed, per year.^13^ We hypothesised that areas with the highest rates of intensive antibiotic prescribing would be associated with the highest rates of UTI resistance.

## Method

### Setting and datasets

This was a cross-sectional, ecological database study in South London’s Lambeth area, comprising 178 Lower layer Super Output Areas (LSOAs), which was undertaken following Strengthening the Reporting of Observational Studies in Epidemiology (STROBE) guidelines. It integrated two datasets: antibiotic prescribing from all GP practices in the borough of Lambeth (*n* = 47) (dataset 1: 2012–2015) and UTI resistance data from two hospitals' microbiology departments (dataset 2: 2013–2015). (The prescribing dataset started 1 year before the resistance set to enable prescribing rate calculations over several years, allowing for a lag between prescribing and resistance.)

Dataset 1: prescribing data originated from a pseudo anonymised primary care database used extensively in previous research (Lambeth DataNet [LDN]).^14^ Data included were details of antibiotic prescriptions issued (name, dosage, and duration of treatment), and demographic and comorbidity details of the patients prescribed antibiotics, per year. Prescribing data were eligible for inclusion if the recipient patient lived in a Lambeth LSOA and was registered with an LDN GP practice on 31 March 2012, 2013, 2014, or 2015.

Dataset 2: data on UTIs were collated by the microbiology department of two hospitals from 2013–2015. These hospitals process urine samples from patients registered with primary care practices that contributed to our prescribing dataset (dataset 1). The UTI data were anonymised patient-level data including age, sex, type of infection, resistance, and/or susceptibility to a panel of five first and second-line antibiotics used for urine infections (amoxicillin, cefalexin, ciprofloxacin, nitrofurantoin, and trimethoprim). Data on all urine samples in the database (excluding samples labelled ‘catheter’) were screened for inclusion. Eligible samples included those that had grown a bacterial organism and were derived from a patient with a Lambeth LSOA postcode. Samples where LSOA was not recorded, were not included in the database (3.5% of original dataset). Age was missing for 0.5% of patients. Sex, type of infection, and resistance and susceptibility were available for all patients.

Both datasets underwent extensive cleaning, formatting, and analysis before merging at the LSOA level, omitting LSOAs with fewer than 10 UTI samples to ensure robust analysis.

### Primary outcome and explanatory variables

The primary outcome was the yearly rate of UTI resistance to any antibiotic, per LSOA. The main explanatory variable was the intensive prescribing rate, per LSOA, which was defined as the percentage of patients receiving ≥5 antibiotic prescriptions over 3 years (as per previous research).^6^ We also defined a subset of intensive prescribing labelled 'very intense', defined as receiving≥4 prescriptions per year. This subset was identified following initial data analysis and reflected approximately 8% of patients in the sample, per year.

The overall volume of prescribed antibiotics, measured in ADQ, was also considered. ADQ is a unit measuring prescribing volume, which represents the average maintenance dose per day for a drug used for its main indication in adults. It is used to compare rates of prescribing across England.^15^

The following co-variates were also included in the combined dataset and reflected the total registered population of LDN per LSOA: age, ethnicity (based on national census 5+1 categories), social deprivation (based on Index of Multiple Deprivation [IMD] 2015) and population prevalence (%) of asthma, chronic obstructive pulmonary diease (COPD), diabetes, dementia, and heart failure. Data for comorbidities were taken from UK primary care Quality and Outcomes Framework (QOF)^16^ statistics for each year and were chosen to reflect long-term conditions for which there is increased susceptibility to infection and subsequent antibiotic prescribing.^17^ There were no missing data for ethnicity, comorbidity, age, and IMD score.

### Statistical analysis

Descriptive statistics were used to describe the primary outcome and prescribing metrics. To estimate the association between the measures of antibiotic prescribing and UTI resistance, we used mixed effects logistic regression with treatment resistance modelled as a binary outcome, and a random component included at LSOA level to account for clustering. All predictors were measured at LSOA level only. Odds ratios and 95% confidence intervals are reported. This paper reports analysis of same year associations, for example, resistance in 2013 and prescribing in 2013, and current year plus 2 preceding years’ associations, for example, resistance in 2014 and prescribing in 2012, 2013, and 2014 (intensive use).

## Results

### Antibiotic prescription and UTI sample data

**Figure 1.**
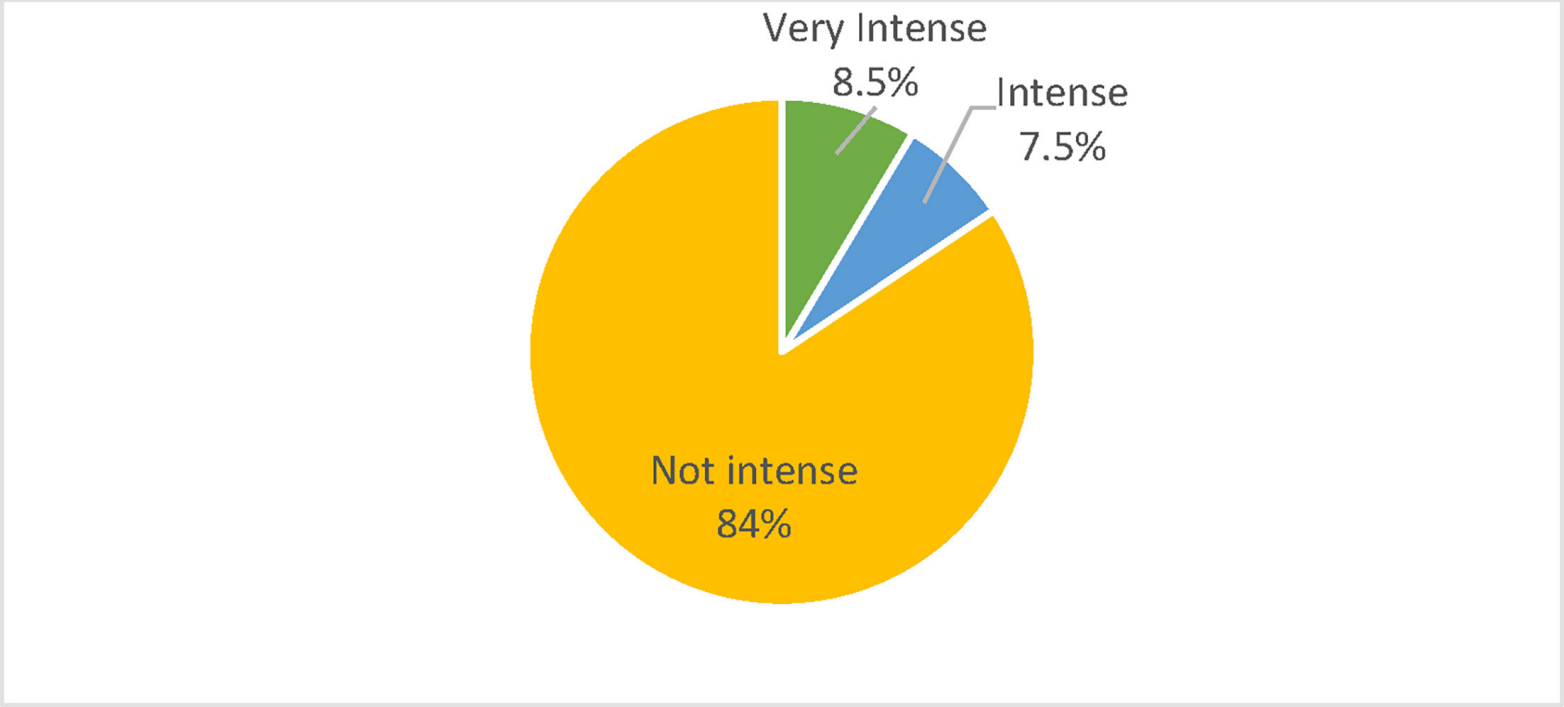
Percentage of antibiotic-prescribed population who received intensive antibiotic prescriptions. Intense: percentage of antibiotic-prescribed population prescribed ≥5 prescriptions over 3 years. Very intense: percentage of antibiotic-prescribed population prescribed ≥4 prescriptions over 1 year. Not intense: percentage of antibiotic-prescribed population prescribed <3 antibiotics within 3 years

**Figure 2.**
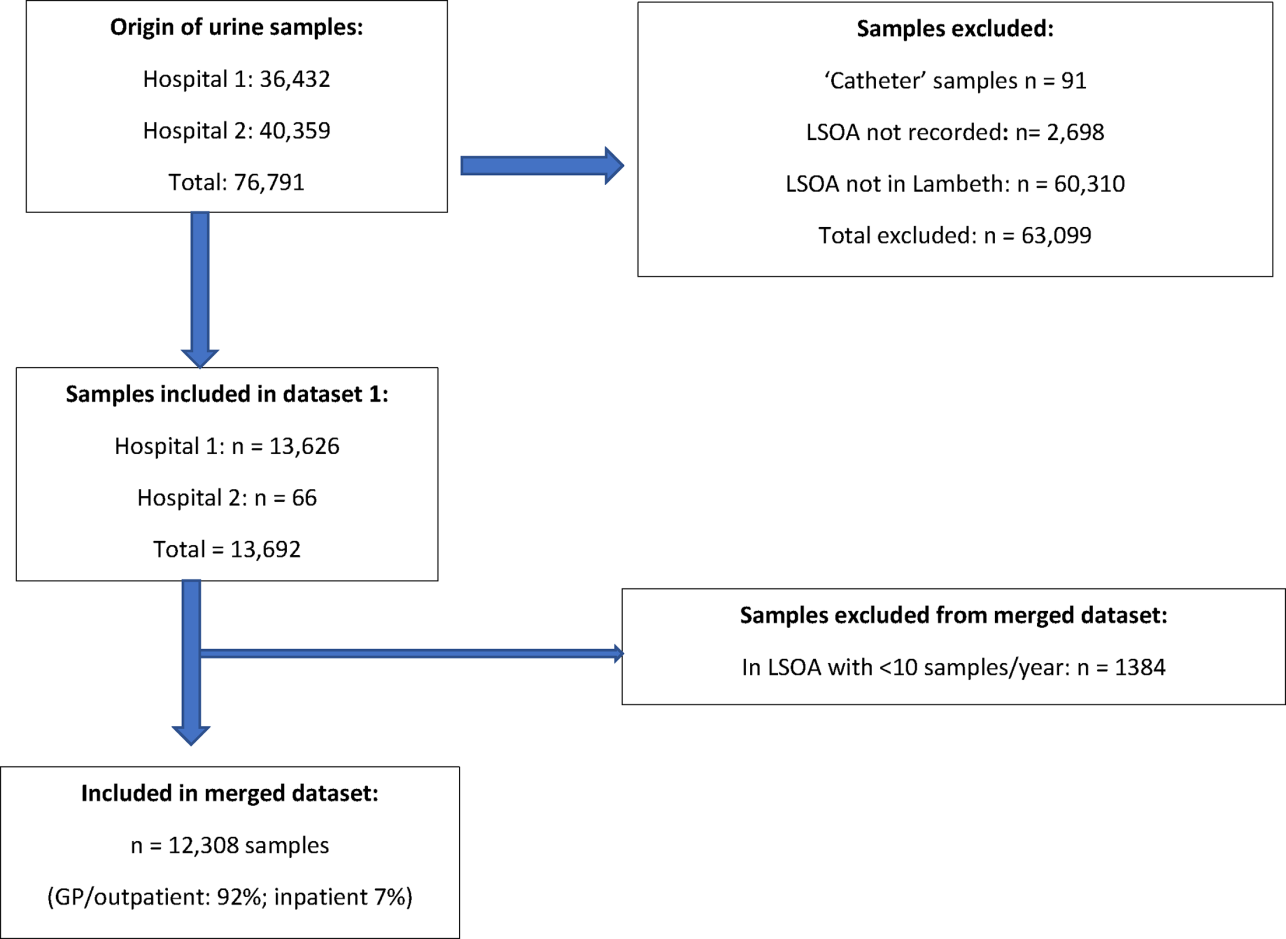
Flowchart for urine samples included in study. LSOA = Lower layer Super Output Area

Following merging of UTI and prescribing data, we included data on 196 513 prescriptions issued to 104 117 patients and resistance data on 12 308 UTI samples. Of the patients prescribed antibiotics, 16% were prescribed intensively (≥ 5 prescriptions in 3 years), receiving almost 30% of prescriptions.We created datasets for each of years 2013, 2014, and 2015, which included the index year resistance data and same year and up to 2 preceding years prescribing data. We had sufficient UTI data (≥10 samples per LSOA) to analyse 53 LSOAs in 2013, 57 in 2014, and 69 in 2015. Summary data are reported in Table 1 and Figure 1 (prescribing) and Figure 2 and Table 2 (resistance).

**Table 1.** Number of prescriptions issued, Average Daily Quantities, and percentage of patients issued repeat antibiotic prescriptions across years 2012–2015

Year (no. of LSOAs)	No. of patients prescribed antibiotics	No. of prescriptions issued	Mean average no. of prescriptions issued per patient per year	ADQ total per year in days	Mean average ADQ per patient per year in days	% patients issued ≥4 prescriptions in 1 year (no. of patients)	% patients issued ≥5 prescriptions over 3 years (no. of patients, years)
**2012** (53)	26 974	47 846	1.8	660 384	24.5	7.9% (2129)	n/a
**2013** (53)	25 668	46 507	1.8	649 513	25.0	8.0% (2047)	n/a
**2014** (57)	25 098	49 091	2.0	688 340	27.4	8.9% (2239)	15.3% (3840) (2012–2014)
**2015** (69)	26 377	53 069	2.0	744 465	28.2	9.0% (2369)	16.7% (4400) (2013–2015)
**Total**	104 117	196 513	1.9	2 742 702	26.3	8.5% (mean)	16% (mean)

ADQ = Average Daily Quantity of all antibiotics prescribed (days). LSOA = Lower layer Super Output Area

**Table 2.** Number of urinary tract infection samples and rate of resistance across years 2013–2015

Year (no. of LSOAs)	**2013** (53)	**2014** (57)	**2015** (69)	Total (134)^a^
**Number of UTIs**	3887	4142	4279	12 308
**Resistant^b^ UTIs**	63.5%	65.6%	65.5%	64.7%

^a^134 unique LSOAs with >10 samples analysed (many LSOAs analysed in more than 1 year). ^b^Resistant to any of five antibiotics (amoxicillin, ciprofloxacin, cefalexin, trimethoprim, and nitrofurantoin). LSOA = Lower layer Super Output Area. UTI = urinary tract Infection

*E. coli* accounted for 80% of infections, for which resistance rates were as follows: amoxicillin: 54.8%; trimethoprim: 37.2%; ciprofloxacin: 12.8%; cefalexin: 8.7%; nitrofurantoin: 2.5% (Supplementary Table S1).

The demographic characteristics of the prescribing data in the merged datasets for each year were similar, and reflective of the total population covered by LDN. Age ranged from 0–105 years; 49% of prescriptions were issued to females. Ethnic diversity mix was broad (White 57%, Black 26%, mixed 8%, Asian 7%, other 2%). Regarding comorbidities, 11% had asthma, 5% diabetes, 1% COPD, 0.6% heart failure, and 0.5% dementia. Mean IMD score was 29.15 (range 7.99–54.05, standard deviation [SD] 9.7), reflecting Lambeth as one of the most deprived boroughs in London (higher IMD scores indicate more severe deprivation). Classes of antibiotics prescribed are summarised in Supplementary Table S2.

### Analysis

Following unadjusted and adjusted logistic regression, we found no significant association between intensive antibiotic prescribing for the same year or for the same year and 2 preceding years and UTI resistance (Table 3). There was also no association with overall ADQ per LSOA (same year) and UTI resistance rates. There were no consistent associations with any other variable studied across the years and increased risk of resistant UTIs.

**Table 3.** The association between community antibiotic prescribing intensity and area level demographic variables or comorbidities with community rates of resistant urinary tract infections

Variables	**2013** UTI resistance per LSOA^a^ Adjusted^b^ OR (95%CI)	**2014** UTI resistance per LSOA^a^ Adjusted^b^ OR (95%CI)	**2015** UTI resistance per LSOA^a^ Adjusted^b^ OR (95% CI)
**Intense prescribing^c^** (**same year and previous 2 years, 2012–2014 and 2013–2015**)	n/a^d^	0.96 (0.92 to 1.00)	1.00 (0.88 to 1.13)
**Very intense prescribing^e^** (**same year**)	1.01 (0.95 to 1.07)	0.94 (0.87 to 1.00)	0.99 (0.94 to 1.00)
**Average Daily Quantity^f^** (**same year**)	1.00 (0.99 to 1.00)	0.99 (0.99 to 1.00)	0.99 (0.99 to 1.00)
**Age 0–4**	0.99 (0.80 to 1.22)	**0.75 (0.57 to 0.98**)	0.92 (0.70 to 1.21)
**Age 5–17**	1.07 (0.95 to 1.20)	0.87 (0.71 to 1.05)	**0.83 (0.70 to 0.98**)
**Age 18–64**	1.08 (0.97 to 1.19)	0.86 (0.72 to 1.04)	0.88 (0.73 to 1.06)
**Age 65–79**	**1.09 (1.03 to 1.16**)	0.87 (0.71 to 1.08)	**0.84 (0.71 to 0.99**)
**Age 80–105**	1.12 (0.89 to 1.39)	1.03 (0.84 to 1.27)	0.83 (0.67 to 1.03)
**White**	0.77 (0.14 to 4.25)	0.77 (0.15 to 3.86)	1.12 (0.19 to 6.32)
**Mixed**	0.76 (0.14 to 4.06)	0.73 (0.15 to 3.55)	1.03 (0.18 to 5.74)
**Black**	0.77 (0.14 to 4.29)	0.79 (0.16 to 4.01)	**1.21 (1.00 to 1.46**)
**Asian**	0.75 (0.14 to 4.16)	0.78 (0.16 to 3.93)	1.23 (0.99 to 1.52)
**Other ethnic group**	0.74 (0.23 to 4.31)	0.77 (0.15 to 4.05)	**1.20 (1.02 to 1.41**)
**IMD score**	0.99 (0.97 to 1.01)	1.00 (0.99 to 1.02)	0.98 (0.96 to 1.00)
**Asthma**	0.97 (0.90 to 1.03)	1.05 (0.99 to 1.14)	0.99 (0.90 to 1.09)
**COPD**	**1.27 (1.01 to 1.60**)	0.95 (0.78 to 1.15)	1.08 (0.83 to 1.40)
**Diabetes**	0.92 (0.78 to 1.61)	**0.80 (0.67 to 0.96**)	1.06 (0.88 to 1.28)
**Dementia**	1.26 (0.98 to 1.62)	0.96 (0.78 to 1.19)	1.11 (0.87 to 1.42)
**Heart failure**	0.78 (0.51 to 1.20)	0.88 (0.55 to 1.41)	1.20 (0.84 to 1.72)

^a^Resistance to any of five panel antibiotics. ^b^Each predicter adjusted for all other listed variables, all variables are percentage of the population who received antibiotics. ^c^Percentage of LSOA antibiotic-receiving population prescribed ≥5 prescriptions over same year and two preceding years 2012–2014; 2013–2015. ^d^Two preceding years of data not available as prescribing dataset started in 2012. ^e^Percentage of LSOA antibiotic-receiving population prescribed ≥4 prescriptions per year. ^f^Average Daily Quantity: unit measuring overall prescribing of antibiotics in days. Bold values indicate value *P*<0.05. CI = confidence interval. COPD = chronic obstructive pulmonary disease. IMD = Index of Multiple Deprivation. LSOA = Lower layer Super Output Area. OR = odds ratio.

## Discussion

### Summary

We found high rates of intensive prescribing (16% of patients) and substantial UTI resistance rates (65%). Despite hypothesising higher rates of intensive prescribing would be associated with increased UTI resistance, we found no such relationship. In the UK, primary care practitioners are encouraged to prescribe responsibly, based on acute clinical presentation. Our study suggests that intensive prescribing for individuals (‘many for the few’) may not be any more detrimental to resistance rates, than antibiotic prescribing overall.

### Strengths and limitations

The study’s use of routine healthcare datasets and its observational nature introduces limitations. Data were not linked at patient level, limiting specific conclusions about individual resistance patterns. Our focus on urban areas with high population densities may limit the applicability of findings to less populated regions. We adjusted for demographic data of those that received prescriptions in our LSOA analysis, a unique strength of this study.

We only included data that originated from a Lambeth LSOA; however, patients may have obtained antibiotic prescriptions not in our sample, from alternative sources, for example, secondary care. We also cannot be sure that a prescription was taken. Additionally, urine samples treated empirically (without culturing) were not available for analysis, but this is reflective of clinical practice guidelines whereby samples are not routinely sent for uncomplicated patients. A unique feature of our study is that we did not exclude hospital samples as the association between community antibiotic prescribing and resistance in samples originating from secondary care has previously been demonstrated.^18,19^

A specific strength of our study is the inclusion of different parameters of antibiotic prescribing, capturing the different ways of quantifying antibiotic prescribing. In 2010, Berrington stated that commonly used indices of antibiotic prescribing do not differentiate between the differences in distribution of antibiotics among individual patients, highlighting how this could affect resistance patterns.^20^ By reviewing existing literature and creating two different metrics of intense prescribing, we distinguished between the frequent and the very frequent users of antibiotics. By using ADQ, a unique tool for measuring prescribing volume in England (which considers the usual adult dose per day), we were able to compare volume of oral antibiotic circulating in each LSOA community, and hence give an indication of ‘overall’ antibiotic prescribing.

The time period chosen of prescribing for the same year and 2 years before ‘resistance’ year, is consistent with previous research studying the relationship at European surveillance level.^21,22^ The delay between changes in rates of antimicrobial prescribing and development of community resistance is however, multifactorial, depending on organism, mechanism of resistance, and population factors.^23^ Although resistance may develop quickly, its decline is often much slower,^24^ with limited evidence regarding time lag from population antibiotic exposure to increased resistance ranging from a few months to years.^22,25^ We evaluated the impact of all antibiotics (as opposed to named antibiotics), as previous research has highlighted how resistance to one antibiotic can be affected by prescribing of another.^26–28^

### Comparison with existing literature

Few studies have specifically addressed antibiotic prescribing intensity, either when considering general prescribing, or its effect on environmental AMR. A US-based outpatient prescriptions study of approximately 20% of the population found, in 1 year, 10% of people received 57% of all antibiotic prescriptions.^7^ A large UK-based cohort study similarly found that nearly 10% of patients received 50% of all prescriptions.^6^ We observed that of nearly 200 000 patients prescribed antibiotics, 16% received nearly 30% of all prescriptions, with over half receiving more than four courses of antibiotics per year. This high level of repeat prescribing to individual patients is concerning, with research showing that individual resistance to an antibiotic may persist for up to 12 months after taking it,^29^ and that repeated individual exposure is related to higher future risk of resistant infections and treatment failure.^30–32^

With regard to the association between intense prescribing and community resistance however, there is limited conflicting evidence. We found no association between intensive ‘any antibiotic’ prescribing over a 3-year period and subsequent resistance. Previously, Olesen *et al* studied US outpatient prescription refills and found that first-time use of specific antibiotics was more strongly associated with resistant specific antibiotic–pathogen combinations than repeated use.^7^ When the same data were re-analysed however, but the focus shifted to ‘any’ antibiotic use, intensive (repeat) use was more often positively associated with resistance to specific antibiotic–pathogen combinations, than first-time use.^8^ This was thought to be owing to reverse causality (the decision to prescribe a specific antibiotic being affected by the choice of antibiotics prescribed previously, weakening the association between repeat prescriptions of the same antibiotic and subsequent resistance). In contrast to our study, the US resistance data were based on a variety of specimens.

We also found no association between overall prescribing (measured by ADQ) and resistance, suggesting resistance to certain bacteria (in this study predominantly *E. coli*) may not be associated with overall prescribing of different antibiotics. Previous research that has found a positive relationship between antibiotic prescribing and UTI resistance has examined specific antibiotic–pathogen combinations.^33,34^

Despite examining multiple variables, we found no consistent independent associations with areas in which there were higher levels of risk factors for developing a resistant UTI. A substantial number of studies suggest that comorbidities are associated with increased antibiotic resistance levels, even after accounting for antibiotic use. Prior antibiotic use, underlying disease, and exposure to invasive procedures during healthcare contact and nursing home residence have been identified as the main human risk factors for developing a resistant infection.^35,36^

### Implications for research and practice

The relationship between antibiotic prescribing and AMR is complex. Our results demonstrated that intense prescribing of multiple antibiotics to individual patients was not crudely associated with increased UTI resistance within the same community, over 1–3 years. From a primary care perspective, this suggests that practitioners should continue to reduce overall prescribing, rather than specifically targeting patients who receive multiple prescriptions. Future research should explore measures to accurately define intensive prescribing, incorporating a measure of antibiotic volume, for example ADQ, as well as considering the impact of intensive prescribing on individual patient resistance rates. Although our data pre-dated COVID-19, the findings remain relevant as prescribing rates have returned to pre-pandemic levels.^37^

## Data

The primary care data used in this project were extracted under the terms of a signed data sharing agreement and as restrictions apply, they are not publicly available.
